# Comparison of Digital Radiography, Computed Tomography, and Magnetic Resonance Imaging Features in Canine Spontaneous Degenerative Stifle Joint Osteoarthritis

**DOI:** 10.3390/ani13050849

**Published:** 2023-02-26

**Authors:** Cheng-Shu Chung, Yi-Ju Tu, Lee-Shuan Lin

**Affiliations:** 1 Department of Veterinary Medicine, College of Veterinary Medicine, National Pingtung University of Science and Technology, Pingtung 91201, Taiwan; 2 Veterinary Medical Teaching Hospital, National Pingtung University of Science and Technology, Pingtung 91201, Taiwan; 3 UniCore Animal Hospital, Taipei City 11494, Taiwan; 4 School of Dentistry, College of Dental Medicine, Kaohsiung Medical University, Kaohsiung 80708, Taiwan; 5 Department of Medical Imaging and Radiological Sciences, College of Health Sciences, Kaohsiung Medical University, Kaohsiung 80708, Taiwan

**Keywords:** canine, stifle joint, computed tomography, digital radiography, magnetic resonance imaging, osteoarthritis

## Abstract

**Simple Summary:**

Canine osteoarthritis (OA) is a common disease in aging dogs and mostly affects the synovial joint. Non-invasive imaging modalities, such as digital radiography (DR) and computed tomography (CT), are often used to diagnose and evaluate the severity of OA. Magnetic resonance imaging (MRI) is the preferred diagnostic method for the assessment of OA, is considered the gold standard in humans, and has been applied in several canine experimental studies. However, the value of MRI in diagnosing spontaneous canine OA and the comparison of different imaging modalities have seldom been addressed. This study compared multiple noninvasive imaging modalities in canine spontaneous stifle OA cases. The results showed that MRI provides the most comprehensive and superior lesion detection sensitivity for ligament, meniscus, cartilage, and synovial effusions. DR provides adequate bony structure information, while CT provides the most delicate images of bony structure lesions. These imaging findings may provide further understanding of the disease and help clinicians draft a more precise treatment plan.

**Abstract:**

Canine stifle joint osteoarthritis (OA) is characterized by damage and degeneration of the articular cartilage and subchondral bone, bony hypertrophy at the margins, and synovial joint membrane changes. Non-invasive imaging modalities, such as digital radiography (DR), computed tomography (CT), and magnetic resonance imaging (MRI), can be used to describe these changes. However, the value of MRI in diagnosing spontaneous canine OA and the comparison of different imaging modalities have seldom been addressed. This study compared multiple noninvasive imaging modalities in canine spontaneous stifle OA cases. Four client-owned dogs with five spontaneously affected OA stifle joints were recruited and underwent DR, CT, and MRI. Information on osteophytes/enthesophytes, ligament/tendon lesions, synovial effusion and membrane thickening, subchondral bone lesions, and meniscal and cartilage lesions were scored and compared. The results showed that MRI provides the most comprehensive and superior lesion detection sensitivity for ligament, meniscus, cartilage, and synovial effusions. DR provides adequate bony structure information, while CT provides the most delicate images of bony structure lesions. These imaging findings may provide further understanding of the disease and help clinicians draft a more precise treatment plan.

## 1. Introduction

Canine osteoarthritis (OA) is a common clinical disorder that affects approximately 20% of skeletally mature dogs [1]. OA is characterized by damage and degeneration of articular cartilage and subchondral bone, bony hypertrophy at the margins, and synovial joint membrane changes, which lead to structural and functional decline of the joint and result in pain and lameness [1].

In clinical practice, noninvasive physical examination and imaging modalities, such as digital radiography (DR), computed tomography (CT), and magnetic resonance imaging (MRI), are commonly used to diagnose and evaluate the severity of OA. DR has the advantages of less space requirements, not necessitating specifically trained staff, less requirements of anesthesia, and lower cost, and hence is mostly applied in diagnosing OA. Common radiographic findings of OA include narrowing of the joint space, subchondral sclerosis, and osteophytosis [2]. However, information on ligament injuries and cartilage defects is limited. CT allows the evaluation of periarticular and articular tissues without superimposition on joint radiography [3]. These images can be constructed with the desired window levels or widths for specific joint anatomic components [2,4]. The disadvantage of CT is that the similar attenuation of complex intra-articular structures of the synovial joint may blur the details. MRI has recently been defined as the ‘‘gold standard’’ in human medicine for synovial joint examination because of the superior soft tissue contrast of the periarticular muscles, tendons, ligaments, and articular cartilage [2]. Signal changes within the bone marrow adjacent to osteoarthritic joints are common findings noted on MRI in humans and the subchondral lesions noted on MRI are considered crucial for determining treatment options for OA patients [5,6]. Nevertheless, the use of MRI in canine OA has not been fully explored. In addition, the settings of MRI for OA are challenging in dogs because the optimal sequences are often geared towards human use, and commercial customized coils for dog limbs are limited.

Previous studies have indicated that spontaneously occurring canine OA and human OA share a similar disease progression phenotype [7,8]. However, the MRI features of canine OA were reported in surgically induced experimental OA models rather than in spontaneous cases [9]. Moreover, a comparison of imaging findings from different imaging modalities in the same patients has also not been reported. Therefore, this study aimed to describe and compare the imaging features of DR, CT, and MRI in spontaneous canine stifle OA cases.

## 2. Materials and Methods

### 2.1. Cases Selection

Adult client-owned dogs with spontaneous chronic (>6 months) stifle OA and associated pain were enrolled in this study. Physical examination, synovial joint fluid analysis, and radiographic findings were used to diagnose osteoarthritis (OA). Dogs with neoplastic joint disease, immune-mediated joint disease, or dogs with a high anesthesia risk (>grade 4 of the American Society of Anesthesiologists) were excluded from this study.

### 2.2. Clinical Lameness Scoring System

Clinical impact of OA on the affected limbs was assessed using an objective clinical scoring system (CLSS, scores: 3–15) (Table 1), which was adopted from a previous study [10]. The CLSS assessed lameness, pain on palpation, and weight-bearing status.

### 2.3. Imaging Exam and Analysis

X-ray examinations were performed using an X-ray machine (MRAD-A50S, Toshiba Medical Systems Corporation, Nasu, Japan) with a digital receptor (CXDI-701c wireless digital radiography system, Canon, Japan). The digital radiographic (DR) images of each affected stifle included caudocranial (CdCr) and mediolateral views. The kVp and mA settings were adjusted according to the radiographic technique chart provided by the manufacturer.

Non-contrast CT was performed using a 64-slice positron emission tomography (PET)/computed tomography (PET/CT) (Discovery 64 Slice PET/CT Scanner, GE Healthcare, New York, NY, USA), with bone plus and standard desired reconstruction algorithm, 150 mAs and 120 kVp, and helical acquisitions with a table pitch factor of 0.516. The CT images were reformatted to soft tissue (window width: 400, window level: 40) and bone (window width: 1500, window level: 450). The dogs were placed in dorsal recumbency on V-shaped foam with a bilateral hindlimb stifle joint positioned at a 90-degree angle.

MRI was performed using a 1.5-T MRI system (Signa 1.5T; GE Healthcare, New York, NY, USA) with a flex coil (GE Signa 1.5 T Flex Coil, GE Healthcare, New York, NY, USA). The dogs were in a laterally recumbent position with the target stifle joint on the non-dependent side at an angle of 135°–145°. The contralateral hind limb was placed at a different angle from the target stifle joint. To reduce artifacts and noise signals, towels and large MRI foam sponges were used to maintain the position of the contralateral hindlimb while the drip lines and capnography sensor were kept away from the target stifle joint. The sequences performed included T1-weighted (T1W), T2-weighted (T2W), proton-density-weighted (PDW), proton-density-weighted fat saturation (PDW fs), and T2 mapping. Standard planes included the sagittal plane, which was oriented parallel to the medial surface of the medial femoral epicondyle; the transverse plane, which was oriented perpendicular to the patellar ligament and sagittal orientation line; and the dorsal plane, which was parallel to the patellar ligament. The scanning parameters are listed in Table 2.

All imaging procedures were performed by residents of veterinary radiology under general anesthesia. Each dog underwent the DR, CT, and MRI examination on the same day. Digital Imaging and Communications in Medicine (DICOM) format was evaluated using diagnostic imaging software (Osirix, Version 9.5.2, Bernex, Switzerland). The original DR, CT, and MRI images were assessed and analyzed by an experienced veterinarian with radiology expertise (L.-S.L.) and a chief resident of veterinary radiology (Y.-J.T.). The final decision was made by consensus.

### 2.4. Articular Lesions Evaluation

Articular lesions of osteophytes and enthesophytes, ligament lesions, synovial effusions, synovial membrane thickening, subchondral lesions, and meniscal lesions were evaluated by grading or presented as “present” or “absent”.

#### 2.4.1. Osteophytes/Enthesophytes

**Figure 1:**
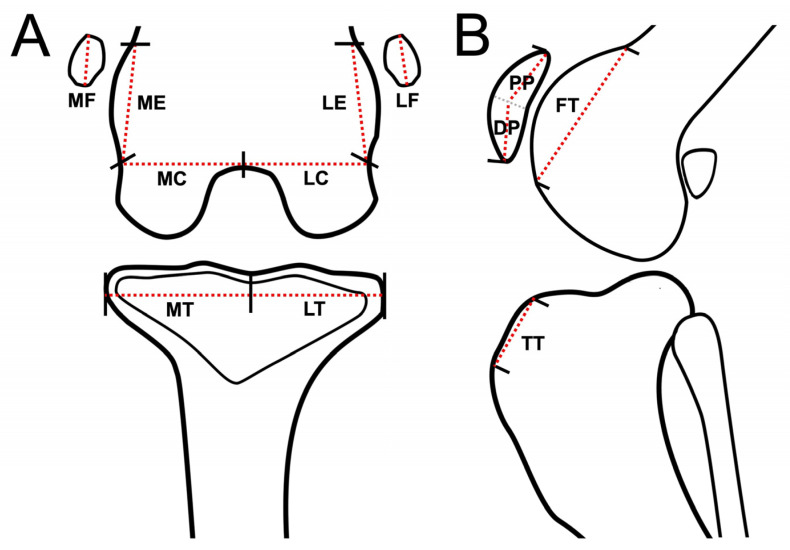
Schematic representation of stifle joint anatomic compartment subregions for evaluation of osteophytes/enthesophytes of each subregion, dorsal plane (MRI and CT) or caudocranial view (DR) (**A**), and sagittal plane (MRI and CT) or lateral view (DR) of stifle joint (**B**). Red dash lines are schematically represented as imaginary anatomic midline (AM) of the stifle joint for each subregion. Stifle joints are divided into 12 subregions for evaluating osteophytes/enthesophytes. Abbreviations: DP: distal patella; FT: femoral trochlear ridges; LC: lateral femoral condyle; LE: lateral femoral epicondyle; LF: lateral fabella; LT: lateral proximal tibia plateau to edge; MC: medial femoral condyle; ME: medial femoral epicondyle; MF: medial fabella; MT: medial proximal tibia plateau to edge; PP: proximal patella; TT: tibial tuberosity.

The osteophytes/enthesophytes in 12 anatomical compartments of the medial femoral epicondyle (ME), lateral femoral epicondyle (LE), medial femoral condyle (MC), lateral femoral condyle (LC), medial proximal tibial plateau to edge (MT), lateral proximal tibial plateau to edge (LT), femoral trochlear ridges (FT), tibial tuberosity (TT), medial fabella (MF), lateral fabella (LF), distal patella (DP), and proximal patella (PP) were evaluated using DR, CT, and MRI (Figure 1) [11]. The sum of the maximum width and height of osteophytes/enthesophytes obtained from the images was divided by the length of the anatomic midlines (AM). The results were then scaled from 0 to 3 (0: none; 1: <25%; 2: 25–50%; 3: >50%).

#### 2.4.2. Ligament/Tendon Lesions

Ligament/tendon lesions were assessed in 6 regions: medial collateral ligament (MCL), lateral collateral ligament (LCL), long digital extensor tendon (LDET), patellar ligament (PL), cranial cruciate ligament (CCL), and caudal cruciate ligament (CdCL). Because the ligament/tendon structure and the lesion cannot be well identified using DR and CT, the severity and incidence of ligament/tendon lesions were only recorded on MRI images and scaled from 0 to 3 (0: normal; 1: partial rupture; 2: partial rupture with thickening or increased peripheral effusion; 3: complete rupture), which was modified from a previous study [12].

#### 2.4.3. Synovial Effusion and Membrane Thickening

**Figure 2:**
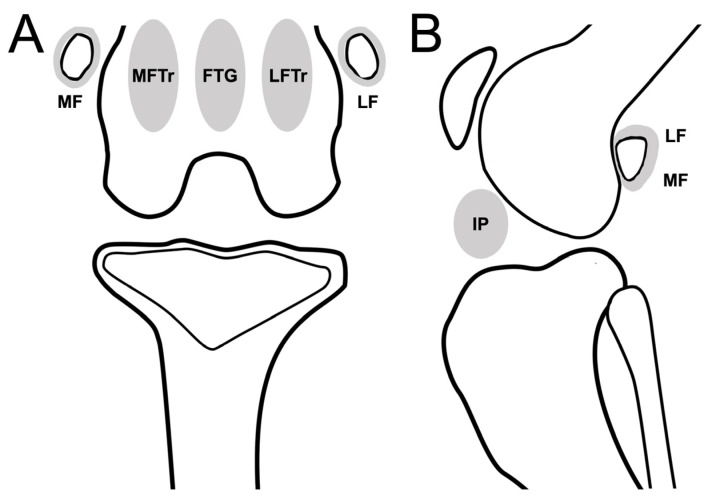
Schematic representation of stifle joint anatomic compartments for evaluating synovial effusion in the dorsal plane (CT or MRI)/caudocranial view (DR) (**A**), and sagittal plane (CT or MRI)/mediolateral view (DR) of stifle joint (**B**). Stifle joint is divided into 6 subregions to evaluate synovial effusion. Abbreviations: FTG, femoral trochlear groove; IP, infrapatellar region; LF, lateral fabella; LFTr, lateral femoral trochlear ridges to epicondyle region; MF, medial fabella; MFTr, medial femoral trochlear ridges to epicondyle region.

The presence of synovial effusion in the infrapatellar region (IP) was recorded in the lateral view of the DR or sagittal planes of the CT and MRI images, and the maximum potential distention of the synovial cavity was scaled from 0 to 3 (0: normal; 1: ≤33%; 2: 33–66%; 3: ≥66%) [13]. The presence of synovial effusion extensions at the MF, LF, medial femoral trochlear ridges to the epicondyle region (MFTr), lateral femoral trochlear ridges to the epicondyle region (LFTr), and femoral trochlear groove (FTG) was recorded on MRI only (Figure 2). In addition, synovial membrane thickening was observed on MRI.

#### 2.4.4. Subchondral Bone Lesions

**Figure 3:**
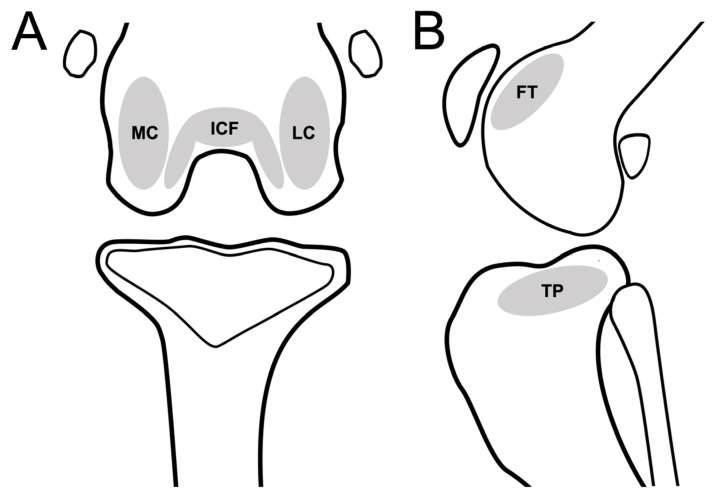
Schematic representation of stifle joint anatomic compartments for evaluating subchondral lesion in the dorsal plane (CT or MRI)/caudocranial view (DR) (**A**), and sagittal plane (CT or MRI)/mediolateral view (DR) of stifle joint (**B**). Stifle joint is divided into 6 subregions to evaluate subchondral lesions. Abbreviations: FT, femoral trochlear; ICF, femoral intercondylar fossa; LC, femoral lateral condyle; MC, femoral medial condyle; TP, tibia plateau.

Subchondral bone defects, cysts, and bone-marrow-edema-like lesions were defined as subchondral bone lesions and evaluated in five anatomical compartments: femoral trochlear (FT), femoral intercondylar fossa (ICF), femoral lateral condyle (LC), femoral medial condyle (MC), and tibial plateau (TP) (Figure 3). The presence of subchondral bone lesions was evaluated using DR, CT, and MRI. Severity was assessed using a modified scale (0: normal; 1: a hypersignal < 1/3 of the surface of the subregion; 2: a hypersignal < 2/3 of the surface of the subregion, 3: a hypersignal > 2/3 of the surface of the subregion) [9].

#### 2.4.5. Meniscal and Cartilage Lesions

**Figure 4:**
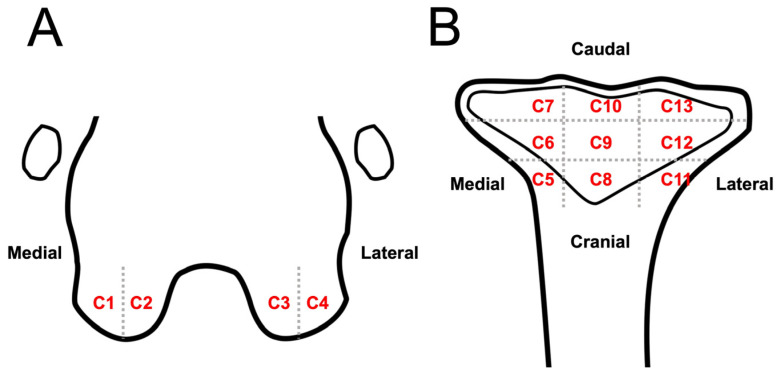
Schematic representation of stifle joint anatomic compartment subregions to evaluate cartilage lesion on MRI images: dorsal plane of the distal femur (**A**) and proximal tibia (**B**). Stifle joint is divided into 13 anatomic subregions to evaluate cartilage lesions: lateral part (A, C1) and medial condyle medial (A, C2); lateral condyle medial (A, C3) and lateral part (A, C4); tibia plateau medial region cranial (B, C5), middle (B, C6), and caudal part (B, C7); tibia plateau middle region cranial (B, C8), middle (B, C9), and caudal part (B, C10); tibia plateau lateral region cranial (B, C11), middle (B, C12), and caudal part (B, C13).

Because meniscal and cartilage lesions cannot be well identified in DR and CT images, these lesions were only evaluated using MRI. Meniscal lesions were evaluated at the medial and lateral menisci and recognized as linear or oval hyperintensity on T2W and PDW images. The severity of meniscal lesions was scaled from 0 to 3 (0, normal; 1, linear or oval core lesion; 2, linear or oval lesion extending to the articular surface; 3, complete rupture) [14]. Cartilage lesions were recognized as heterogeneous signals of cartilage, irregular cartilage contour, and cartilage defects/rupture. The presence of cartilage lesions was recorded in 13 anatomical compartments and numbered from C1-C13: two compartments of MC (C1, C2) and LC (C3, C4), and nine compartments of the tibial plateau (C5–C13) (Figure 4). T2 relaxation times for evaluating cartilage lesions at the medial femoral condyle, lateral femoral condyle, medial tibial plateau, and lateral tibial plateau were calculated by manually drawing the region of interest (ROI) on color-coded T2 mapping images with a cartilage thickness > 0.5 mm (>1 pixel). The results were recorded and compared with those of a previous study [15].

### 2.5. Statistical Analysis

Data were statistically analyzed using commercially available software (GraphPad Prism, ver. 7.00, San Diego, CA, USA). Descriptive statistics (mean, median, and percentage) were applied for continuous variables (age, body weight, and T2 relaxation time) and ordinal variables (CLSS and scores for each grading articular lesion). Categorical variables (signalments and affect joints) are presented in tabular form. Comparisons of scales obtained from DR, CT, and MRI images were performed using the Kruskal–Wallis test followed by Dunn’s multiple comparisons test.

## 3. Results

### 3.1. Animals

Four dogs (three Beagles and one Labrador retriever) with five stifle joints (three left and two right) met the inclusion criteria and were recruited. Three dogs were spayed females and one dog was an intact male. The median age of the dogs was 15 years (range, 10–17 years). The median weight was 11.7 kg (range, 7.8–28.0 kg). Hindlimb lameness was noted in all dogs, and the median CLSS score was 9.0 (range, 8–10).

### 3.2. Articular Lesions

#### 3.2.1. Osteophytes/Enthesophytes

**Figure 5:**
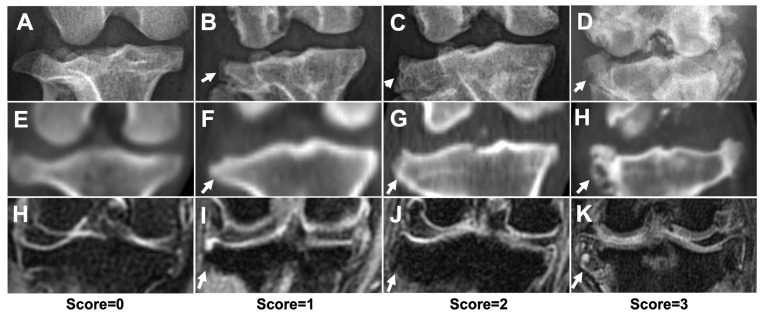
The severity of osteophytes/enthesophytes of stifle joint using different imaging modalities. Representative DR (**A**–**D**), CT (**E**–**H**), and MRI (**H**–**K**) images, with score = 0 (**A**,**E**,**H**), score = 1 (**B**,**F**,**I**), score = 2 (**C**,**G**,**J**), and score = 3 (**D**,**H**,**K**). White arrows indicated osteophytes/enthesophytes formation at lateral proximal tibia edges. The score = 0 images were acquired from a dachshund which was not included in this study.

**Figure 6:**
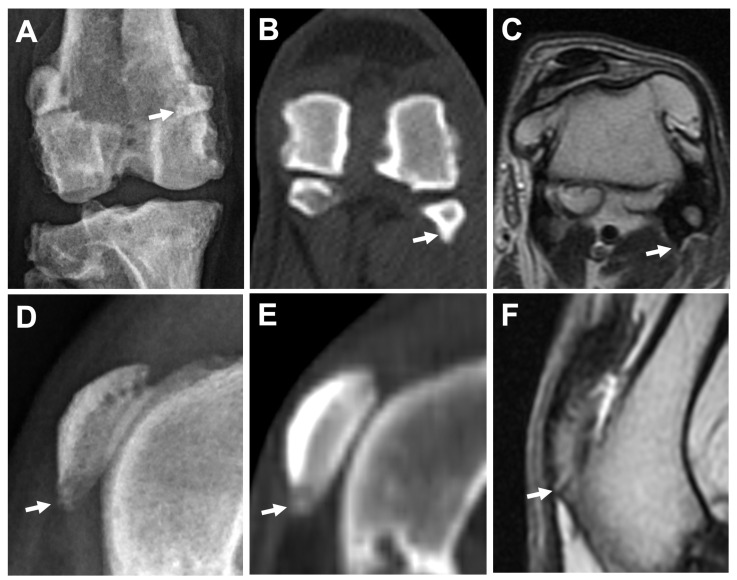
Comparison of osteophytes/enthesophytes at fabella using different imaging modalities in the same leg. Caudocranial view of DR (**A**), bone window of CT (transverse plane) (**B**), and T2W MRI (transverse plane) (**C**) images. Enthesophytes/osteophytes at lateral fabella (arrows) are superimposed with the distal femur on DR image (**A**), but can be easily identified on CT image (**B**), while exhibiting similar hypointense signal with the attached femoropatellar ligament on MRI image (**C**). Mediolateral view of DR (**D**), and sagittal plane of CT (**E**) and MRI (**F**) images in the same leg. A hyperintense projection of the distal patella (arrow), which can be seen on MRI (**F**) with a score = 2, is graded score = 1 and score = 0 on DR (**D**) and CT (**E**) images, respectively.

Representative images of the severity of osteophytes/enthesophytes in the stifle joint are shown in Figure 5. The incidences and scores of osteophytes and enthesophytes in each anatomical compartment are listed in Table 3. In the ME, LE, MC, LC, MT, and LT subregions, osteophytes/enthesophytes were identified in all joints by all modalities. In the FT subregion, osteophytes and enthesophytes could not be identified in only one joint on DR. The tibial tuberosity subregion was the most uncommon site for the osteophytes/enthesophytes, and they were only found in one joint (20%) by different modalities. In the medial and lateral fabella subregions, CT (100%) had a higher sensitivity than DR (60%) and MRI (60%) (Figure 6A–C). In the distal patella and proximal patella subregions, MRI (100%) had higher sensitivity than DR (60%) and CT (40%) (DP, PP) (Figure 6D–F). No significant differences in osteophytes/enthesophytes lesion scores at different anatomic subregions among DR, CT, and MRI were noted.

#### 3.2.2. Ligament/Tendon Lesions

**Figure 7:**
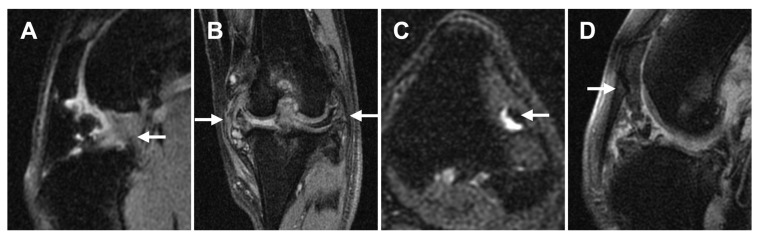
Representative MRI images of ligament/tendon lesions at each site. (**A**): Mixed intensities are seen in both CCL and CdCL (sagittal plane, PDW fs). (**B**): Both MCL and LCL are mildly thickened and have hyperintensities within the ligament (dorsal plane, PDW fs). (**C**): Focal hyperintensity is noted within the LDET with fluid accumulation surrounding the tendon (transverse plane, T2W fs). (**D**): Mixed intensity is noted adjacent to the distal patella at PL (sagittal plane, PDW fs). Abbreviations: CCL, cranial cruciate ligament; CdCL, caudal cruciate ligament; LCL, lateral collateral ligament; LDET, long digital extensor tendon; MCL, medial collateral ligament; PL, patellar ligament.

Ligament/tendon lesions were mostly noted in the CCL (5/5 [100%]), CdCL (5/5 [100%]), and LCL (5/5 [100%]) regions, followed by the MCL (3/5 [60%]), LDET (1/5 [20%]), and PL (1/5 [20%]) on MRI. The median (range) ligamental/tendon scores were 1 (0–2) for MCL, 1 (1–2) for LCL, 0 (0–1) for LDET, 0 (0–1) for PL, 1 (1–3) for CCL, and 1 (1–3) for CdCL. Representative images of the ligament/tendon ligament lesions are shown in Figure 7.

#### 3.2.3. Synovial Effusion and Membrane Thickening

**Figure 8:**
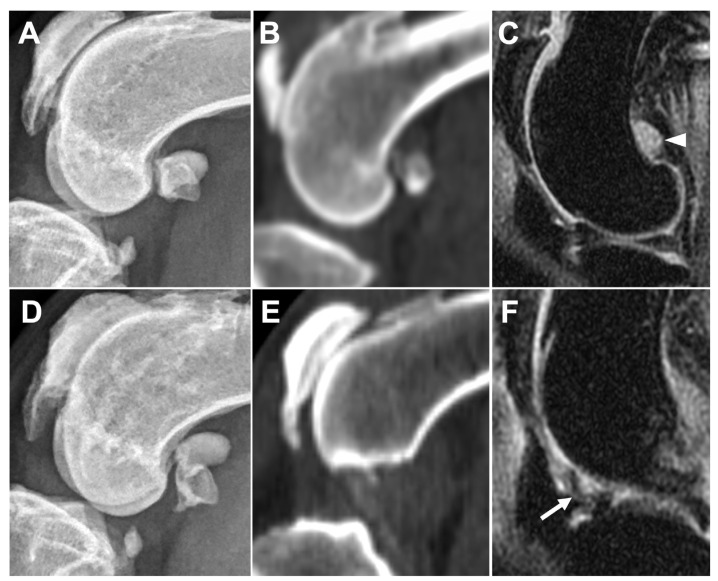
Comparison of medial fabella synovial effusion on DR (**A**), CT (**B**), and MRI (**C**), and infrapatellar synovial membrane thickening on DR (**D**), CT (**E**), and MRI (**F**) in the same leg. Obvious synovial effusion (arrowhead) is seen at the medial fabella region on MRI image (**C**) but cannot be observed on DR (**A**) or CT (**B**) images. Synovial membrane thickening is seen as low intense signal inside the synovial cavity (arrow) on MRI (**F**) but cannot be observed on DR (**D**) or CT (**E**) images.

The presence of synovial effusion at the IP was recorded on DR (4/5 [80%]), CT (5/5 [100%]), and MRI (5/5 [100%]), with median (range) synovial effusion extension scores 2 (0–2), 1 (1–3), and 1 (1–2), respectively, which were not significantly different between the modalities (*p* = 0.8519). In contrast, an extension of synovial effusion was identified on MRI in MF (4/5 [80%]), LF (3/5 [60%]), MFTr (3/5 [60%]), LFTr (1/5 [20%]), and FTG (1/5 [20%]). The MF was the most remarkable extension site of the synovial effusion (Figure 8 A–C). Synovial membrane thickening was identified only in the IP region of all joints on MRI (Figure 8D–F).

#### 3.2.4. Subchondral Bone Lesions

**Figure 9:**
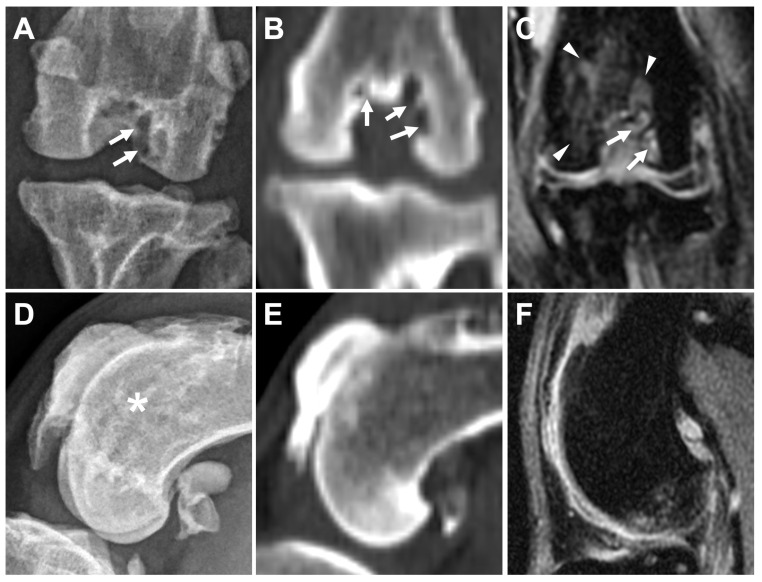
Comparison of subchondral bone defects, cysts, and bone-marrow-edema-like lesions of caudocranial view in caudocranial view on DR (**A**), bone window on CT (dorsal plane) (**B**), and PDW fs (dorsal plane) on MRI (**C**) in the same leg. Subchondral bone defects and cysts are mostly seen in two areas on DR (**A**, arrow), in three areas on CT (**B**, arrow), and in two areas on MRI (**A**, arrow). Subchondral bone marrow edema-like lesions surrounding the intertrochlear fossa are only identified on MRI (**C,** arrowheads). Subchondral bone defect-like lesion is suspected at the femoral trochlear region on DR image (**D**, asterisk), but not identified on CT (**E**) nor MRI (**F**).

Subchondral bone lesions were noted in all modalities (Figure 9). The incidence and scores of subchondral bone lesions in the different compartments on DR, CT, and MRI are listed in Table 4. ICF was the most common site of subchondral bone lesions, which could be noted in most joints, except for one joint on the DR images. In contrast, lesions were less commonly seen in FT, and only two joints were identified on DR images. Although CT and MRI provided similar sensitivities in detecting lesions, CT exhibited higher sensitivity in TP. In addition, no significant differences in subchondral bone lesion scores in each compartment were observed among the different modalities.

#### 3.2.5. Meniscal and Cartilage Lesions

**Figure 10:**
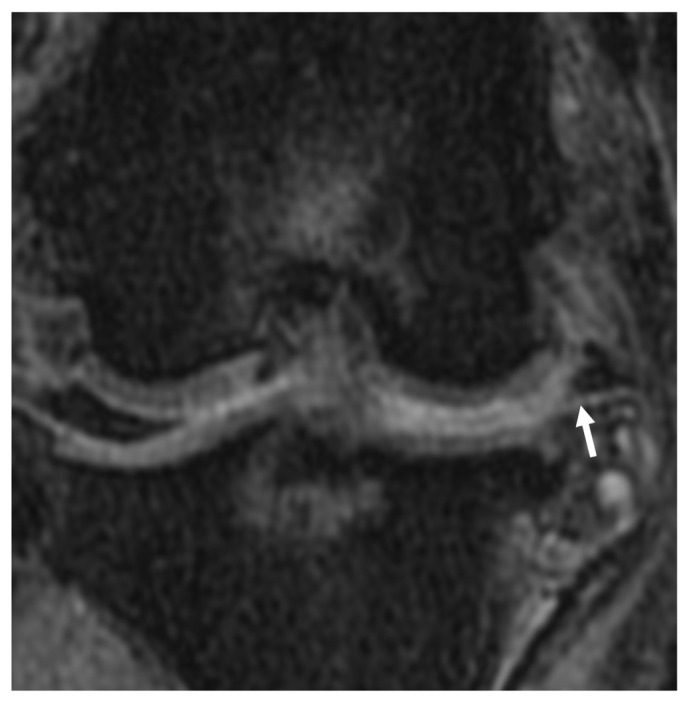
Meniscal maceration lesion noted on the dorsal plane, PDW fs MRI image. Chronic degenerative meniscal changes resulting the separation of the meniscus into constituent fragments (arrow). Synovial effusion accumulation is noted between the femoral condyle and tibial plateau, where the meniscus normally exists.

Medial meniscal lesions were noted in two joints (2/5 [40%]), with scores of 1 and 3, respectively. Except for linear or oval core lesions, a meniscal maceration lesion, which is a specific term used in MRI to describe chronic degenerative meniscal lesions causing meniscal wasting and becoming separated fragments, was noted in one joint (Figure 10). A lateral meniscal lesion was only seen in one joint, which was encountered together with the medial meniscal lesion, with a score of 2. Cartilage lesions were noted in almost all subregions of all joints except for two subregions (C5 and C8) in one stifle joint. The T2 relaxation time of the cartilage lesions was generally lower than that of previously published data (Table 5).

## 4. Discussion

This study recruited canine patients with naturally occurring chronic stifle OA with moderate lameness and evaluated DR, CT, and MRI imaging findings. Although similar clinical performances were noted in these patients, the individual associated soft and bony structural changes may differ, and the results could be altered using different modalities. Currently, various treatment options are available for canine OA, including surgery, rehabilitation, regeneration therapy, and nutritional supplementation. Detailed knowledge from these imaging modalities is essential for developing customized treatment for OA.

Our results indicated that osteophytes/enthesophytes could form in almost all anatomical compartments of the stifle joint in chronic OA, except for the tibial tuberosity. This is considered an acceptable result, since the tibial tuberosity is an extra-articular structure and may not be involved in the OA process. In addition, the osteophytes/enthesophytes at the femoral epicondyle (ME, LE), femoral condyle (MC, LC), and fabella (MT, LT) were the most evident and could be identified by all modalities. CT and MRI may be more sensitive than DR at femoral trochlear ridges (FT) because the superimposition of osteophytes, enthesophytes, and distal femur can be avoided.

In the fabella subregions (MF and LF), it may be difficult to identify osteophytes and enthesophytes by using DR and MRI. Severe osteophytes in the distal femur may superimpose on the fabella in the CdCr view, and bilateral fabellae may superimpose on each other in the lateral view. Moreover, multiple connective tissues may superimpose on each other at this site, such as the femoropatellar ligament and the origin of the gastrocnemius on DR images. These connective tissues also present hypointense signals on MRI; thus, they can sometimes be confounded by osteophytes and enthesophytes. In contrast, CT has the following advantages: multiple plane images; imaging processing by three-dimensional CT rendering and reconstruction; and the superior ability in differentiating the soft and bony tissues, allowing better identification of the osteophytes and enthesophytes better than the other modalities at the fabella site.

In the patellar region (DP, PP), MRI provided the highest sensitivity in detecting osteophytes and enthesophytes. The texture of osteophytes and enthesophytes at an early stage is similar to cartilage and appears radiolucent on DR or CT images, whereas the osteophytes and enthesophytes around the patella in the early stage may present hyperintense signals compared with cortical bone on MRI owing to incomplete mineralization [16,17]. This enables early detection of osteophytes and enthesophytes on MRI. In addition, the superimposition of incompletely mineralized osteophytes on DR images may provide better identification than CT.

It is challenging to evaluate ligament/tendon lesions using non-invasive imaging examinations, especially DR and CT. Nevertheless, MRI is useful for diagnosing ligamental/tendon lesions. In this study, various severities of ligament/tendon lesions were identified in the six ligaments and tendons around the stifle joints. CCL, CdCL, and LCL lesions were the major ligament lesions observed in all joints. CCL disease is the most common cause of hindlimb lameness in dogs, and progressive stifle OA will develop [18,19]. Although the exact pathogenesis of canine CCL disease remains controversial, aging changes resulting in degeneration of the CCL may be associated with programmed cell death, inflammatory cell infiltration, and extracellular matrix degeneration, and may contribute to the event [19,20,21,22]. Therefore, it is not surprising that CCL lesions were notable. In addition, the CdCL, which is close to the CCL in the same space, may share the same process of degeneration. This might explain the same incidence and severity of CCL and CdCL lesions here, even though injury of the CdCL was less commonly noted in dogs encountering traumatic processes. Interestingly, the LCL also revealed notable changes simultaneously with CCL and CdCL. Since the LCL can be injured when the tibia is pushed outward or excessively internally rotated, lesions of the LCL may result from excessive tension after CCL injury [23,24]. In contrast, the LDET and PL were the most common sites of ligament lesions. Unlike ligaments, tendons are responsible for the extension or flexion of joints, rather than the maintenance of joint stability. As a result, the lesions in the LDEL and PL were not notable and may not be related to the OA process.

Synovial effusion at the IP subregion, which was strongly correlated with chronic OA, was found in all modalities. Consistent results have also been reported in experimental canine OA models and working dogs [11,25]. Although all three modalities provided a high detection rate, CT and MRI were more sensitive than DR for detecting synovial effusion in the IP region. The extension of synovial fluid was mostly noted around the fabella in our cases, similar to the results reported in dogs undergoing experimental surgery after 26 weeks [11]. This may indicate that the fabella region, including the femorofabellar joint and joint capsule, is also important in OA processing. In contrast, synovial extension is least seen in the trochlear region (FTG), and it is thought that the presence of the patellar bone in the trochlear groove may limit the extension. In addition, thickening of the synovial joint capsule in the IP region was also identified in all joints on MRI. These results indicated that MRI exhibited high sensitivity for fluid signals and synovial membrane structures in stifle joints, which were not well identified on DR or CT.

The subchondral lesion or bone marrow lesion may indicate inflammation, edema, and ischemic condition of the bone marrow [26,27,28], which presents as T2W and PDW hyperintense lesions on MRI. Nevertheless, DR and CT provide details of bony structures, such as subchondral bone defects or cysts. Our results revealed that subchondral lesions at the FT were only noted on DR images. Combining the CT and MRI findings of the same joint, these lesions resulted from the superimposition of regional osteophytes with trochlear ridges rather than “true” subchondral lesions (Figure 9). Therefore, the findings of DR imaging, used to detect subchondral lesions in the FT subregion, should be interpreted with caution. Although subchondral bone plays a crucial role in the initiation and progression of human OA [29], the clinical values still can not be identified here due to the chronic OA stage and single time point for assessment. However, a previous study demonstrated that the abnormal bone metabolism was detected before subchondral bone lesion formatted in human OA, and the sodium fluoride positron emission tomography/CT coregistered with MRI could serve as a feasible molecular imaging modality to assess early post-traumatic OA [30].

Meniscal lesions were observed less commonly in this study. Previous studies revealed that the degenerative change and cranial cruciate ligament rupture could be associated with meniscal lesions [31,32]. However, the underlying causes could not be identified due to limited information on illness history.

Cartilage lesions are common and extensive in canine stifle OA. Although a previous study showed that MRI could evaluate the morphology of articular cartilage [33], only the integrity of the cartilage could be documented in our study. Although a higher magnetic field MRI may be required to evaluate the details of stifle joint cartilage in small-to-middle-breed dogs, severe cartilage defects could still be detected using 1.5T MRI.

A recent study applied T2 relaxation time to determine changes in cartilage composition in normal beagles [15]. T2 relaxation time reflects the water composition change in articular cartilage and provides early detection of articular cartilage injury and subsequent mineralization. The increase in T2 relaxation time could be used to detect cartilage damage in dogs, whereas the decrease may be related to severe OA or articular cartilage mineralization in humans [34,35]. The T2 relaxation time in our study was generally lower than previous results in normal beagle stifle joints [15]. The profound cartilage lesions with severe OA observed in our cases may explain these changes.

Our study had several limitations. The primary limitation is the lack of comparison between imaging findings with arthroscopy and histological examination because all cases were client-owned clinical cases. Another limitation of our study is its cross-sectional design with a small sample size of clinical OA cases, making it difficult to investigate the etiology of OA lesions. Further longitudinal studies including more dogs and patients with different severities of OA are warranted.

## 5. Conclusions

In conclusion, comparing the imaging modalities for canine stifle OA, DR provides adequate bony structure information, the shortest examination time, and the least budget consumption; however, most soft tissue and cartilage lesions could not be seen. In addition, the superimposition of abundant osteophytes and enthesophytes may have misled the results. CT provides the most delicate bony structure lesion information with a moderate examination time. However, articular soft tissue lesions cannot be obtained without invasive techniques (e.g., contrast arthrography), and a higher cost than DR is required. MRI provides images of the superior ligament, meniscus, cartilage, and synovial effusion with adequate bony structure information. Although the disadvantages of long anesthesia time during the examination and high cost are still of concern, MRI provides the most comprehensive joint images compared to other non-invasive imaging modalities used in this study, and these findings provide detailed information on stifle OA.

## Figures and Tables

**Table 1:** Clinical lameness scoring system. Adapted with the permission from Ref. [10]. 2013, the Korean Society of Veterinary Science.

Criterion	Grade	Clinical Evaluation
Lameness	1	Walks normally
	2	Slightly lame when walking
	3	Moderately lame when walking
	4	Severely lame when walking
	5	Reluctant to rise and will not walk more than five paces
Pain on palpation	1	None
	2	Mild signs; dog turns head in recognition
	3	Moderate signs; dog pulls limb away
	4	Severe signs; dog vocalizes or becomes aggressive
	5	Dog will not allow palpation
Weight-bearing	1	Equal on all limbs standing and walking
	2	Normal standing; favors affect limb when walking
	3	Partial weight-bearing standing and walking
	4	Partial weight-bearing standing; non-weight-bearing walking
	5	Non-weight-bearing standing and walking

**Table 2:** The MRI scanning parameters of the canine stifle joint.

Sequence	Plane	TR (msec)	TE (msec)	NEX	ET	Slice (mm)	Gap (mm)	FOV (cm)	Matrix
T1 FSE	Sagittal	605	Min Full	5	3	2.2	0	10	256 × 200
PD FSE	Sagittal	2500	34	4	7	2.2	0	10	224 × 200
T2 FSE	Sagittal	3000	60	5	16	2.2	0	10	248 × 188
PD FSE FS	Sagittal	2500	34	4	7	2.2	0	10	256 × 200
3D FSPGR FS	Sagittal	21.5	Min Full	3	n/a	2	0	10	256 × 200
T2 FSE FS	Transverse	3865	60	5	16	2.5	0	10	240 × 200
T2 FSE	Transverse	3825	60	5	16	2.5	0	10	240 × 200
PD FSE	Dorsal	2500	34	4	7	2.2	0	10	252 × 200
T2 FSE	Dorsal	3045	60	5	16	2.2	0	10	252 × 200
PD FSE FS	Dorsal	2500	34	4	7	2.2	0	10	200 × 200

Abbreviations: ET, echo train length; FOV, field of view; NEX, number of excitations; TE, echo time; TR, repetition time.

**Table 3:** Comparison of the osteophytes/enthesophytes lesion on DR, CT, and MRI images at different anatomic subregions.

Compartments	Modality	Incidence	Scales	*p*-Value	Compartments	Modality	Incidence	Scales	*p*-Value
ME	DR	5/5 (100%)	2 (1–3)	>0.999	FT	DR	4/5 (80%)	2 (0–3)	>0.999
	CT	5/5 (100%)	3 (1–3)			CT	5/5 (100%)	3 (1–3)	
	MRI	5/5 (100%)	3 (1–3)			MRI	5/5 (100%)	3 (1–3)	
LE	DR	5/5 (100%)	2 (1–3)	>0.999	TT	DR	1/5 (20%)	0 (0–2)	>0.999
	CT	5/5 (100%)	3 (1–3)			CT	1/5 (20%)	0 (0–3)	
	MRI	5/5 (100%)	3 (1–3)			MRI	1/5 (20%)	0 (0–3)	
MC	DR	5/5 (100%)	2 (1–3)	>0.999	MF	DR	3/5 (60%)	2 (0–3)	>0.999
	CT	5/5 (100%)	2 (1–3)			CT	5/5 (100%)	2 (1–2)	
	MRI	5/5 (100%)	3 (1–3)			MRI	3/5 (60%)	2 (0–3)	
LC	DR	5/5 (100%)	2 (1–3)	>0.999	LF	DR	3/5 (60%)	2 (0–3)	>0.999
	CT	5/5 (100%)	2 (1–3)			CT	5/5 (100%)	2 (1–2)	
	MRI	5/5 (100%)	3 (1–3)			MRI	3/5 (60%)	2 (0–3)	
MT	DR	5/5 (100%)	2 (1–3)	>0.999	DP	DR	4/5 (80%)	2 (0–3)	>0.999
	CT	5/5 (100%)	2 (1–3)			CT	4/5 (80%)	3 (0–3)	
	MRI	5/5 (100%)	2 (1–3)			MRI	5/5 (100%)	3 (1–3)	
LT	DR	5/5 (100%)	2 (1–3)	>0.999	PP	DR	3/5 (60%)	1 (0–3)	0.2222
	CT	5/5 (100%)	2 (1–3)			CT	2/5 (40%)	0 (0–3)	
	MRI	5/5 (100%)	2 (1–3)			MRI	5/5 (100%)	1 (1–3)	

Abbreviations: DP, distal patella; FT, femoral trochlear ridges; LC, lateral femoral condyle; LE, lateral femoral epicondyle; LF, lateral fabella; LT, lateral proximal tibial plateau to edge; MC, medial femoral condyle; ME, medial femoral epicondyle; MF, medial fabella; MT, medial proximal tibial plateau to edge; TT, tibial tuberosity; PP, proximal patella.

**Table 4:** Comparison of the subchondral bone lesions on DR, CT, and MRI images at different anatomic subregions.

Compartments	Modality	Incidence		Scores		*p*-Value
FT	DR	2/5	(40%)	0	(0–1)	0.3333
	CT	0/5	(0%)	0	(0–0)	
	MRI	0/5	(0%)	0	(0–0)	
ICF	DR	4/5	(80%)	1	(0–1)	0.3333
	CT	5/5	(100%)	1	(1–1)	
	MRI	5/5	(100%)	1	(1–3)	
LC	DR	1/5	(20%)	0	(0–1)	0.6667
	CT	1/5	(20%)	0	(0–1)	
	MRI	2/5	(40%)	0	(0–2)	
MC	DR	1/5	(20%)	0	(0–1)	0.5185
	CT	3/5	(60%)	0	(0–1)	
	MRI	3/5	(60%)	1	(0–2)	
TP	DR	1/5	(20%)	0	(0–1)	0.5556
	CT	3/5	(60%)	1	(0–1)	
	MRI	1/5	(20%)	0	(0–2)	

Abbreviations: FT, femoral trochlear ridges; ICF, femoral intercondylar fossa; LC, femoral lateral condyle; MC, femoral medial condyle; TP, tibia plateau.

**Table 5:** Results of cartilage MRI T2 relaxation time.

Cartilage Lesions	Relaxation Time (ms)	Reference [15]
MF	44.5 (range 40.0–52.3)	70.2 (range: 57.9–87.9)
LF	43.8 (range 39.2–54.1)	57.5 (range: 46.8–66.9)
MT	43.7 (range 37.4–55.2)	65.0 (range: 52.0–92.0)
LT	42.4 (range 39.7–45.9)	57.0 (range: 49.0–66.2)

Abbreviations: LF, lateral femoral condyle; LT, lateral tibia plateau; MF, medial femoral condyle; MT, medial tibia plateau.

## Data Availability

The data presented in this study are available upon request from the corresponding author.
